# Charting elimination in the pandemic: a SARS-CoV-2 serosurvey of blood donors in New Zealand

**DOI:** 10.1017/S0950268821001643

**Published:** 2021-07-30

**Authors:** Lauren H. Carlton, Tiffany Chen, Alana L. Whitcombe, Reuben McGregor, Greg Scheurich, Campbell R. Sheen, James M. Dickson, Chris Bullen, Annie Chiang, Daniel J. Exeter, Janine Paynter, Michael G. Baker, Richard Charlewood, Nicole J. Moreland

**Affiliations:** 1 School of Medical Sciences, The University of Auckland, Auckland, New Zealand; 2 The New Zealand Blood Service, Auckland, New Zealand; 3 Callaghan Innovation, Christchurch, New Zealand; 4 School of Biological Sciences, The University of Auckland, Auckland, New Zealand; 5 School of Population Health, The University of Auckland, Auckland, New Zealand; 6 Department of Public Health, University of Otago, Wellington, New Zealand

**Keywords:** COVID-19, elimination, New Zealand, receptor binding domain, SARS-CoV-2, seroprevalence, serosurvey, Spike

## Abstract

New Zealand has a strategy of eliminating SARS-CoV-2 that has resulted in a low incidence of reported coronavirus-19 disease (COVID-19). The aim of this study was to describe the spread of SARS-CoV-2 in New Zealand via a nationwide serosurvey of blood donors. Samples (*n* = 9806) were collected over a month-long period (3 December 2020–6 January 2021) from donors aged 16–88 years. The sample population was geographically spread, covering 16 of 20 district health board regions. A series of Spike-based immunoassays were utilised, and the serological testing algorithm was optimised for specificity given New Zealand is a low prevalence setting. Eighteen samples were seropositive for SARS-CoV-2 antibodies, six of which were retrospectively matched to previously confirmed COVID-19 cases. A further four were from donors that travelled to settings with a high risk of SARS-CoV-2 exposure, suggesting likely infection outside New Zealand. The remaining eight seropositive samples were from seven different district health regions for a true seroprevalence estimate, adjusted for test sensitivity and specificity, of 0.103% (95% confidence interval, 0.09–0.12%). The very low seroprevalence is consistent with limited undetected community transmission and provides robust, serological evidence to support New Zealand's successful elimination strategy for COVID-19.

New Zealand has a strategy of eliminating SARS-CoV-2 that has resulted in a low incidence of coronavirus-19 disease (COVID-19). The first case was reported on 26 February 2020, and the country entered a strict nationwide lockdown one month later for 49 days [1]. Through rigorous border control and managed isolation and quarantine facilities for new arrivals, New Zealand has since remained largely COVID-19 free. Globally, serological surveillance has been utilised throughout the pandemic to define the cumulative incidence, including estimations of missed cases and/or asymptomatic infection. Due to lockdowns and movement restrictions, blood donors have been used as a sentinel population in many settings [2, 3]. The aim of this study was to describe the spread of SARS-CoV-2 in New Zealand via a blood donor serosurvey. Though the pandemic response has been highly effective, PCR testing was initially restricted due to limited diagnostic reagents [4] and there have been occasional border incursions and small community outbreaks, including a cluster in August 2020 with no identified link to the border.

Samples were collected by the New Zealand Blood Service via nine static collection centres and 36 mobile collection services over a 4-week period (3 December 2020–6 January 2021) from individuals aged 16–88 years. Duplicates were removed, leaving 9806 samples for analysis. Compared with the 2018 New Zealand census, participants were more likely to be aged 40–59 years (43.3% *vs.* 25.9%) and of European ethnicity (77.8% *vs.* 61.0%) but had a similar proportion of females (49.1% *vs.* 50.7%) and were geographically spread with 16 of 20 district health board regions represented (Table 1 and Supplementary Appendix). This study was assessed by the Health and Disability Ethics Committee, and additional consent was not required (21/CEN/21).

**Table 1.** Demographics of the blood donors, 2018 Census population and COVID-19 cases in New Zealand

	Blood donors		2018 Census population		COVID-19 cases	
	*n*	%	*n*	%	*n*	%
Total	9806	100.00	4 793 361	100.00	2190	100.00
Sex						
Male	4990	50.89	2 364 315	49.30	1037	47.35
Female	4816	49.11	2 429 046	50.70	1153	52.65
Prioritised ethnicity						
Māori	710	7.24	777 195	16.20	192	8.77
Pacific Island	192	1.96	314 202	6.60	184	8.40
Asian	921	9.39	705 384	14.70	393	17.95
MELAA	144	1.47	68 283	1.40	73	3.33
European/other^a^	7631	77.82	2 924 175	61.00	1337	61.05
Unknown	208	2.12	4122	0.10	11	0.50
Age						
0–14 years	0	0.00	927 102	19.34	177	8.08
15–19 years^b^	238	2.43	308 304	6.40	110	5.02
20–29 years	1754	17.89	681 963	14.20	520	23.76
30–39 years	1870	19.07	624 363	13.00	398	18.14
40–49 years	2053	20.94	619 641	12.90	309	14.12
50–59 years	2196	22.39	623 445	13.00	320	14.62
60–69 years	1437	14.65	510 327	10.60	222	10.14
70+ years	258	2.63	498 216	10.40	134	6.12
District Health Board (DHB)						
Northland	200	2.04	179 007	3.70	28	1.28
Auckland	1478	15.07	467 595	9.80	226	10.32
Waitemata	613	6.25	586 329	12.20	297	13.57
Counties Manukau	494	5.04	537 633	11.20	217	9.91
Waikato	1096	11.18	405 555	8.50	194	8.86
Taranaki	285	2.91	117 684	2.50	16	0.73
Lakes	282	2.88	109 080	2.30	16	0.73
Bay of Plenty	550	5.61	240 117	5.00	48	2.19
Tairawhiti	0	0.00	47 520	1.00	4	0.18
Hawkes Bay	51	0.52	166 287	3.50	44	2.01
Mid central	792	8.08	174 993	3.70	32	1.46
Whanganui	0	0.00	64 599	1.30	9	0.41
Wairarapa	51	0.52	45 327	0.90	8	0.37
Hutt Valley	214	2.18	148 509	3.10	24	1.10
Capital and Coast	1082	11.03	303 957	6.30	96	4.39
Nelson Marlborough	0	0.00	150 528	3.10	49	2.24
West Coast	0	0.00	31 578	0.70	5	0.23
Canterbury	1774	18.09	539 628	11.30	168	7.67
South Canterbury	113	1.15	58 977	1.20	17	0.78
Southern	731	7.45	324 387	6.80	216	9.87
Unknown	0	0.00	94 071	2.00	0	0.00
Managed Isolation and Quarantine (MIQ)						
–	0	0.00	0	0.00	476	21.70

MELAA, Middle Eastern, Latin American and African.

The New Zealand blood service donations were collected between the 3rd of December 2020 and the 6th of January 2021. Of the 9806 individuals, 9771 are blood donors and 35 are living tissue and stem cell donors. All donors must be free of illness and weigh >50 kg. Notable travel and a history SARS-CoV-2 infection (or contact with a positive case) are recorded prior to collection. The health board region provided for the donors is based on donation location. Demographics for COVID-19 cases were obtained from the New Zealand Ministry of Health and include probable and confirmed infections up to and including the 6th of January 2021. The most recent New Zealand census took place in March 2018. Priority ethnicity is reported as defined by the New Zealand Department of Statistics.

a ‘Other’ comprises of *n* = 28 (0.20%) of the blood donor population, and *n* = 51 447 (1.10%) of the New Zealand census population.

b All blood donors are at least 16 years of age.

Antibodies to the Spike (S) protein and receptor-binding domain (RBD) persist for many months after infection, compared with antibodies to the nucleocapsid (N) protein [5, 6], providing a rationale for the use of S protein-based assays in serosurveys. The overall serological testing algorithm was optimised for specificity given the low number of reported COVID-19 cases in New Zealand (2190 as of 6 January 2021) and the associated period prevalence of 0.04%, which limits the positive predictive value of tests with reduced specificity [7]. Samples were first screened with a widely used and well-validated two-step ELISA that comprises a single point dilution assay against the RBD followed by titration against trimeric S protein (Supplementary Appendix) [8, 9]. Samples above the cut-off were tested on two further immunoassays – the EuroImmun SARS-CoV-2 IgG ELISA (EuroImmun AG, Lübeck, Germany) and the cPass surrogate Viral Neutralisation Test (sVNT) (GenScript, New Jersey, USA) and the values deemed seropositive if above the cut-off on both commercial assays. Sensitivity and specificity for these assays were determined by Receiver Operator Characteristic (ROC) curves based on previous analyses (413 pre-pandemic negatives, 99 PCR confirmed cases) (Supplementary Appendix) [9, 10].

Of the 9806 samples, 18 were positive for both Spike IgG (EuroImmun) and antibodies that block the RBD-hACE-2 interaction (sVNT), with the values highly correlated (Pearson *r* 0.7993, *P* < 0.0001) (Fig. 1). Further analysis of the 18 seropositive samples with a multiplex bead-based assay that detects antibody isotype reactivity to RBD, S and N proteins [5] revealed a pattern consistent with infections that occurred weeks or months prior; a dominance of RBD and S protein IgG with few samples positive for N protein IgG, nor IgA or IgM against any of the three antigens (Fig. 1). Within these 18 seropositive samples, six were retrospectively matched to donors with previously confirmed SARS-CoV-2 infections. That all confirmed cases were detected supports the rationale of the testing algorithm applied. A further four seropositive samples were from donors with 2020 travel history in settings with a high risk of SARS-CoV-2 exposure (UK and Europe), suggesting likely infection outside New Zealand. The remaining eight seropositive samples were from seven different district health regions, giving a crude seroprevalence estimate of 0.082% (95% confidence intervals (CI) 0.035–0.16%). Applying the Rogan–Gladen estimate with the Lang–Reiczigel CI method to account for test sensitivity and specificity resulted in a true seroprevalence estimate of 0.103% (95% CI 0.09–0.12%) (Supplementary Appendix). This corresponds to an infection to case ratio of 2.3, based on notified cases on 6 January 2021, suggesting some undiagnosed infections have occurred. However, this ratio needs to be interpreted with caution for two reasons. First, the limitations of the sampling population including the absence of those aged <16 years of age, and a lower proportion of those of Māori and Pacific ethnicity compared with the 2018 census population. Second, the extremely small number of seropositive donors makes extrapolation unreliable and this also precludes any subgroup analysis.

**Fig. 1.**
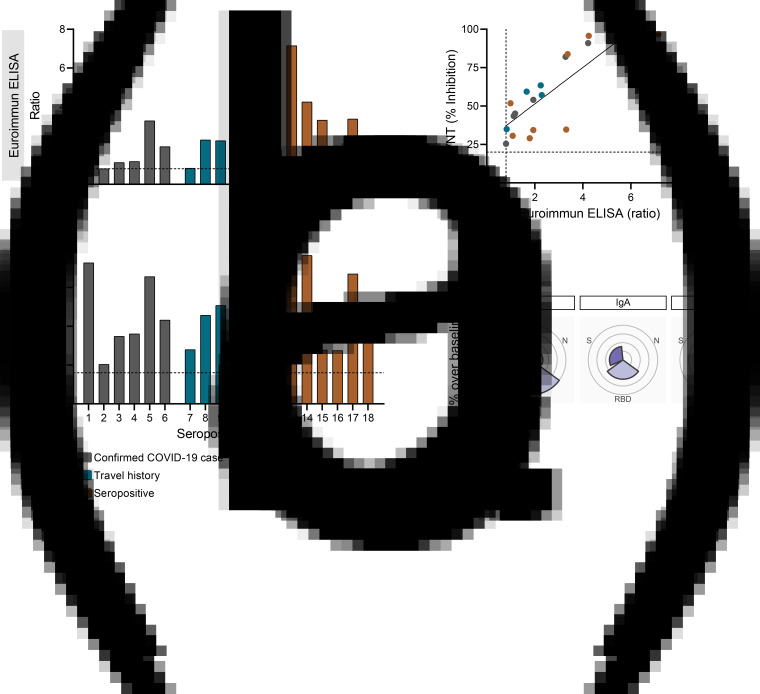
Antibody characteristics of the seropositive donors (*n* = 18). (a) Seropositivity was confirmed by EuroImmun S1 IgG (top) and the surrogate Viral Neutralisation Test (sVNT, bottom). Six donors had PCR-confirmed SARS-CoV-2 infection (dark grey), four had relevant travel history (dark turquoise) and eight were identified in this study (orange). The manufacturer cut-offs are shown (black dotted line). (b) Pearson correlation of sVNT and the Euroimmun IgG ELISA (*n* = 18). (c) Rose plot showing the percentage of seropositive donors over baseline for IgG, IgA and IgM antibodies against the RBD, Spike (S) and nucleocapsid (N) proteins determined using a multi-plex Luminex bead assay.

The very low seroprevalence of SARS-CoV-2 infection in New Zealand implies that undetected community transmission has been limited. This seroprevalence is broadly similar to a recent study conducted in the low prevalence city of Sydney in Australia [3], and markedly lower than estimates of >10% from serosurveys in Europe and North America where the pandemic has been poorly controlled (https://serotracker.com). This study provides robust, serological evidence of New Zealand's successful elimination strategy ahead of vaccine roll-out and highlights the value of a nationwide blood donor service to monitor viral spread during the pandemic.

## Supplementary material

For supplementary material accompanying this paper visit http://dx.doi.org/10.1017/S0950268821001643. click here to view supplementary material

## Data

Data, in addition to those available in the Supplementary information, are available from the authors on request.
